# Steric repulsion and supra­molecular assemblies *via* a two-dimensional plate by C—H⋯O hydrogen bonds in two closely related 2-(benzo­furan-2-yl)-2-oxoethyl benzoates

**DOI:** 10.1107/S2056989017010556

**Published:** 2017-07-21

**Authors:** Li Yee Then, C. S. Chidan Kumar, Huey Chong Kwong, Yip-Foo Win, Siau Hui Mah, Ching Kheng Quah, S. Naveen, Ismail Warad

**Affiliations:** a X-ray Crystallography Unit, School of Physics, Universiti Sains Malaysia, 11800 USM, Penang, Malaysia; b Department of Engineering Chemistry, Vidya Vikas Institute of Engineering & Technology, Visvesvaraya Technological University, Alanahally, Mysuru 570 028, Karnataka, India; c School of Chemical Sciences, Universiti Sains Malaysia, Penang 11800 USM, Malaysia; d Department of Chemical Science, Faculty of Science, Universiti Tunku Abdul Rahman, Perak Campus, Jalan Universiti, Bandar Barat, Perak, Malaysia; e School of Biosciences, Taylor’s University, Lakeside Campus, 47500 Subang Jaya, Selangor, Malaysia; f Institution of Excellence, University of Mysore, Manasagangotri, Mysuru 570 006, India; g Department of Chemistry, Science College, An-Najah National University, PO Box 7, Nablus, West Bank, Palestinian Territories

**Keywords:** crystal structure, benzo­furan, inter­molecular inter­action, functional group

## Abstract

2-(Benzo­furan-2-yl)-2-oxoethyl 2-chloro­benzoate, C_17_H_11_ClO_4_ (I), and 2-(benzo­furan-2-yl)-2-oxoethyl 2-meth­oxy­benzoate, C_18_H_14_O_5_ (II), were synthesized under mild conditions. Their chemical and mol­ecular structures were analyzed by spectroscopic and single-crystal X-ray diffraction studies, respectively. These compounds possess different *ortho*-substituted functional groups on their phenyl rings, thus experiencing extra steric repulsion force within their mol­ecules as the substituent changes from 2-chloro (I) to 2-meth­oxy (II). The crystal packing of compound (I) depends on weak inter­molecular hydrogen bonds and π–π inter­actions. Mol­ecules are related by inversion into centrosymmetric dimers *via* C—H⋯O hydrogen bonds, and further strengthened by π–π inter­actions between furan rings. Conversely, mol­ecules in compound (II) are linked into alternating dimeric chains propagating along the [101] direction, which develop into a two-dimensional plate through extensive inter­molecular hydrogen bonds. These plates are further stabilized by π–π and C—H⋯π inter­actions.

## Abstract 2

The title compounds are constructed from a benzo­furan ring and an *ortho*-substituted phenyl ring connected by a carbonyl-connecting bridge. The structural conformation of the compounds are under substantial influence of steric repulsion. In the crystals, the mol­ecules are connected by C—H⋯O hydrogen bonds and π–π inter­actions [with extra C—H⋯π inter­action for compound (II)].

## Chemical context   

Benzo­furans are an important class of heterocyclic compounds with fused benzene and furan rings. The benzo­furan nucleus has been widely used as the building block for various biologically active compounds due to its broad range of pharmacological properties (Swamy *et al.*, 2015 ▸; Zhou *et al.*, 2010 ▸; Rangaswamy *et al.*, 2012 ▸). Benzo­furan derivatives, especially with substituents at their C-2 position, are commonly found in natural products and synthetic compounds. Several reviews describing the biological potential of these scaffolds acting as anti­oxidant (Chand *et al.*, 2017 ▸), anti­microbial (Hiremathad *et al.*, 2015 ▸), anti­cancer and anti­viral (Khanam & Shamsuzzaman, 2015 ▸) agents have been published. Encouraged by previous studies, we are herein reporting the synthesis, spectroscopic studies and structure determination of 2-(benzo­furan-2-yl)-2-oxoethyl 2-chloro­benzoate (I) and 2-(benzo­furan-2-yl)-2-oxoethyl 2-meth­oxy­benzoate (II).
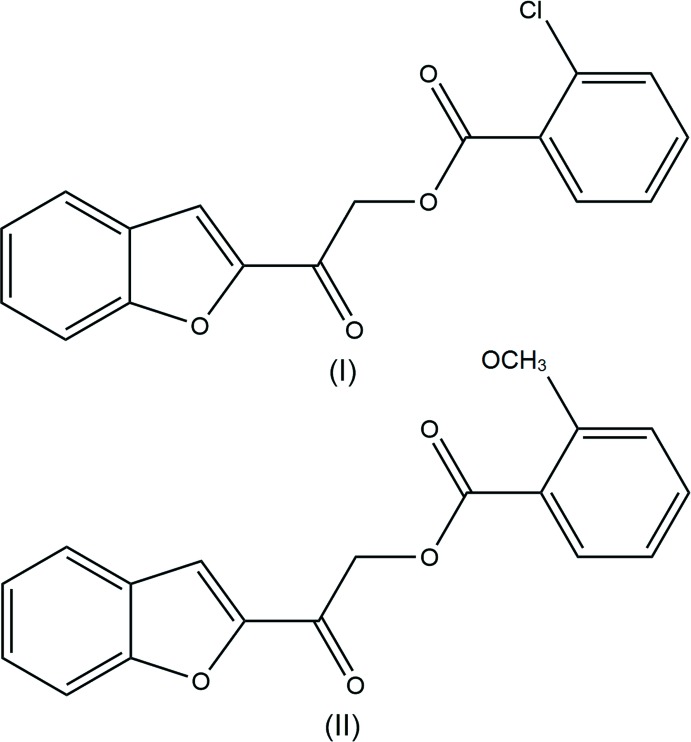


## Structural commentary   

**Figure 1:**
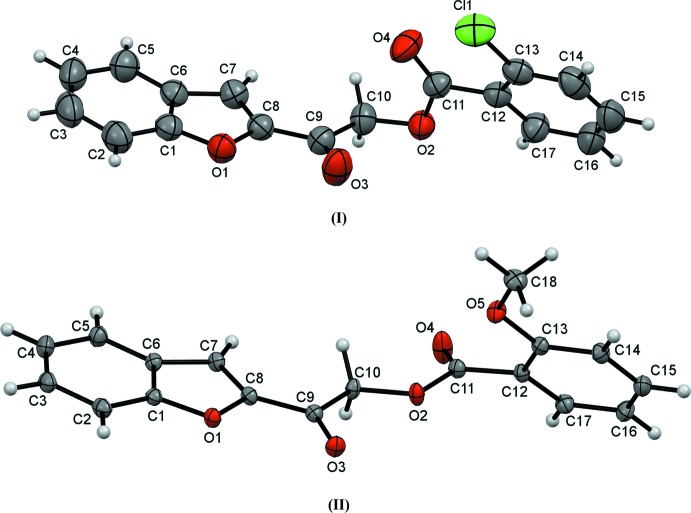
The structures of (I) and (II), showing 50% probability displacement ellipsoids and the atomic labelling scheme.

**Figure 2:**
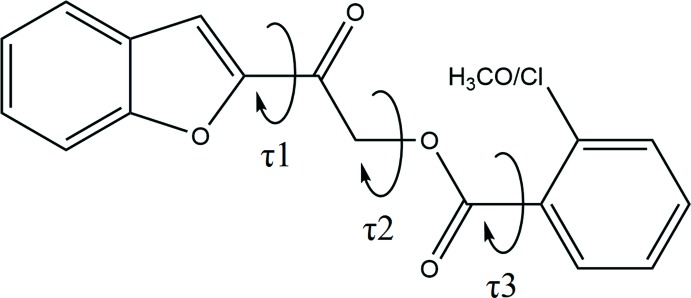
General chemical diagram showing the torsion angles τ1, τ2 and τ3.

The mol­ecular structure of the title compounds (Fig. 1 ▸) contain two ring systems, which are the benzo­furan and the *ortho*-substituted [chloro- for (I) and meth­oxy- for (II)] phenyl rings, inter­connected by a C—C(=O)—O—C(=O) connecting bridge. The unique mol­ecular conformations of compounds (I) and (II) can be characterized by three torsion angles, *i.e.* τ1 (O1—C8—C9—O3), τ2 (C9—C10—O2—C11) and τ3 (O4—C11—C12—C13) respectively (Fig. 2 ▸). The torsion angle τ1 for both structures is approximately 0°, signifying the coplanarity between their benzo­furan ring and the adjacent carbonyl group at the connecting bridge. As for the torsion angle between the two carbonyl groups, τ2, compound (I) exhibits a *syn-clinal* conformation [C9—C10—O2—C11 = −76.19 (17)°] whereas compound (II) adopts an *anti-periplanar* conformation [C9—C10—O2—C11 = −173.51 (9)°]. Likewise, owing to the *ortho*-substitution of the functional group at their phenyl rings, both studied compounds experience steric repulsion between their substituent and adjacent carbonyl groups, which can influence torsion angle τ3. Greater steric repulsion force is observed between carbonyl group and meth­oxy groups [O4—C11—C12—C13 = 123.09 (14)° for compound (II)] than with the chlorine atom [O4—C11—C12—C13 = 22.0 (3)° for compound (I)] (Then *et al.*, 2017 ▸).

## Supra­molecular features   

**Figure 3:**
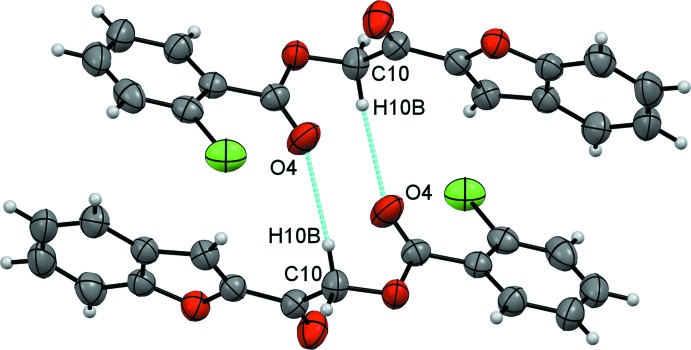
The dimeric structure of compound (I) formed by two adjacent inversion-related mol­ecules.

**Figure 4:**
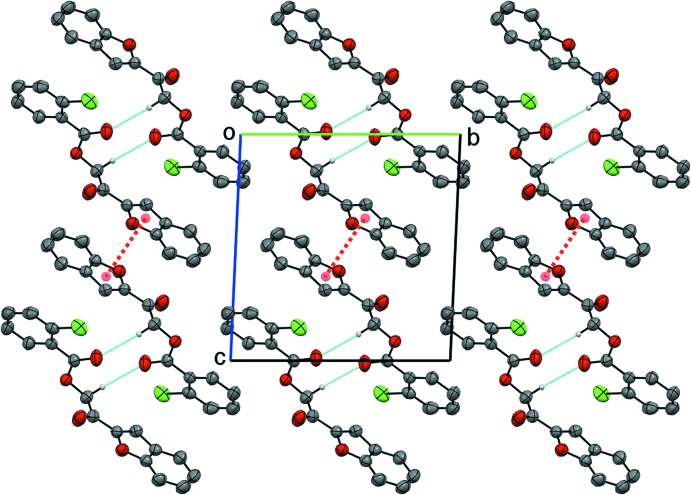
The crystal packing of compound (I), showing hydrogen bonds (cyan dotted lines) and π–π inter­actions (red dotted lines).

The crystal packing of compound (I) features weak inter­molecular hydrogen bonds (Table 1 ▸) and π–π inter­actions. Two inversion-related mol­ecules are joined to form a centrosymmetric dimer by a pair of weak inter­molecular C10—H10*B*⋯O4 hydrogen bonds, generating an 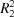
(10) graph-set motif (Fig. 3 ▸). These dimers are further consolidated by π–π inter­actions, involving two face-to-face related furan rings, distanced by 3.6623 (11) Å, propagating along the [001] direction (Fig. 4 ▸) with symmetry code −*x* + 1, −*y* + 1, −*z* + 1.

**Figure 5:**
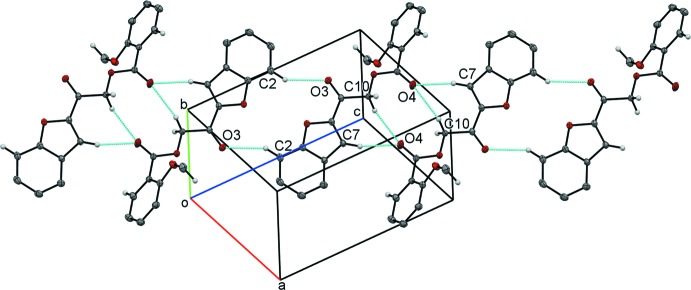
Inter­molecular hydrogen bonds joining mol­ecules into an endless chain in compound (II).

**Figure 6:**
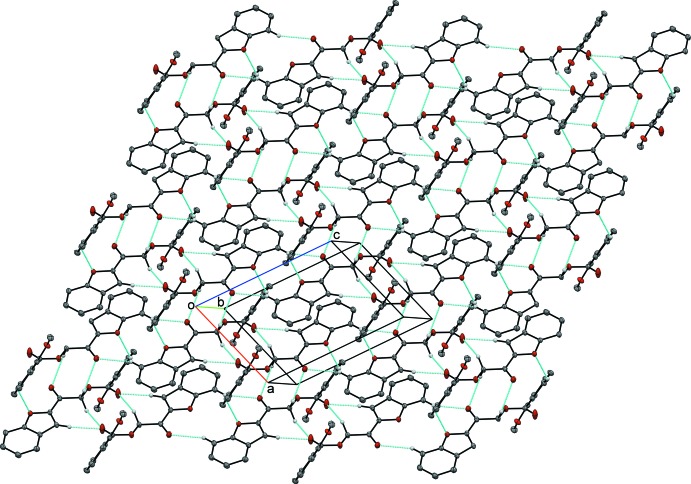
Inter­molecular inter­actions in compound (II), showing the two-dimensional plate parallel to the *ac* plane.

**Figure 7:**
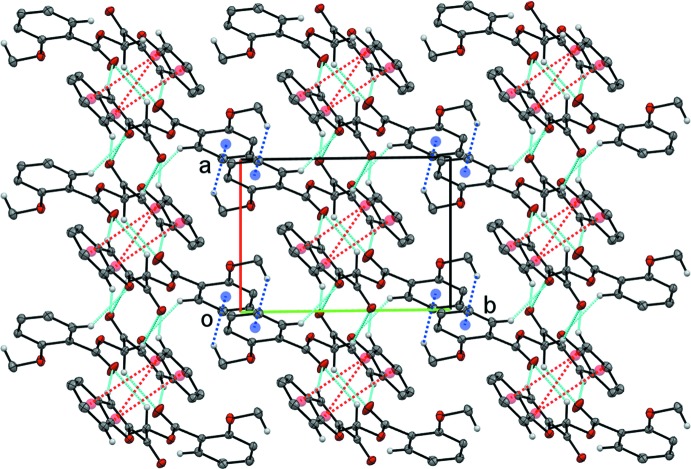
Extensive inter­molecular inter­actions in compound (II), showing hydrogen bonds (cyan dotted lines), C—H⋯π inter­actions (blue dotted lines) and π–π inter­actions (red dotted lines).

Contrasting with compound (I), compound (II) is assembled by extensive inter­molecular inter­actions (Table 2 ▸). Mol­ecules are linked into inversion dimer–dimer chains through weak C2—H2*A*⋯O3, C10—H10*B*⋯O4 and C7—H7*A*⋯O4 hydrogen bonds, propagating along the [101] direction (Fig. 5 ▸). The centrosymmetric dimer formed by the C2—H2*A*⋯O3 hydrogen-bond pairs generates an 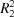
(14) ring motif. On the other hand, atom O4 serves as a bifurcated acceptor in the 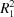
(7) motif and yet, participates in a second 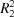
(10) ring motif. These dimer–dimer chains are further expanded by C10—H10*A*⋯O3 and C17—H17*A*⋯O1 hydrogen bonds through inversion to build a two-dimensional plate parallel to the *ac*-plane (Fig. 6 ▸). Within these plates, two kinds of π–π inter­actions further stabilize the crystal packing: these inter­actions are between furan rings [centroid–centroid separation: 3.4402 (7) Å; symmetry code: −*x* + 1, −*y* + 1, −*z* + 1] and between a furan ring and a benzene ring [centroid–centroid separation: 3.6088 (7) Å; symmetry code: −*x* + 1, −*y* + 1, −*z* + 1]. In addition, neighboring plates are inter­connected *via* C—H⋯π inter­actions involving the substituted meth­oxy group and an adjacent phenyl ring (Fig. 7 ▸).

## Database survey   

A search of the Cambridge Structural Database (Groom *et al.*, 2016 ▸) by using 2-(1-benzo­furan-2-yl)-2-oxoethyl benzoate as reference skeleton has revealed five benzo­furan structures (Kumar *et al.*, 2015 ▸) related to the title compounds, *i.e.* ITAXUY, ITAYAF, ITAYEJ, ITAYIN and ITAYOT. The mol­ecular structures of these compounds differ only at their substituted phenyl rings, especially compound (I) with ITAYAF and compound (II) with ITAYIN, which have the same substituents at altered positions. By looking at their torsion angles at the C—C(=O)—O—C(=O) carbonyl connecting bridge, compound (I) was found to exhibit a *syn*-clinal conformation similar to ITAXUY, ITAYEJ and ITAYIN (τ2 ranges from 75 to 80°) whereas compound (II) shows an *anti*-periplanar conformation as do ITAYAF and ITAYOT (ranging from 163 to 180°).

## Synthesis and crystallization   

The title compounds were synthesized by dissolving a mixture of 1-(benzo­furan-2-yl)-2-bromo­ethan-1-one (1 mmol) with 2-chloro­benzoic acid (1 mmol) for compound (I) and 2-meth­oxy­benzoic acid (1 mmol) for compound (II) in *N*,*N*-di­methyl­formamide (8 ml). The solution was stirred for about two h at room temperature in the presence of a catalytic amount of anhydrous potassium carbonate and the progress was monitored by thin-layer chromatography (TLC). Once the reaction was complete, the resultant mixture was poured into a beaker of ice cooled water which gave a precipitate. The precipitate obtained was then filtered, washed with distilled water and dried. Finally, pure crystals suitable for X-ray analysis were obtained by slow evaporation using a suitable solvent.

**2-(Benzo­furan-2-yl)-2-oxoethyl 2-chloro­benzoate (I):**

Solvent used to grow crystals: acetone; yield: 79%; m.p. 366–368 K. ^1^H NMR (500 MHz, CDCl_3_) in ppm: δ 8.086–8.070 (*d*, 1H, *J* = 7.9 Hz, ^17^CH), 7.773–7.757 (*d*, 1H, *J* = 7.9 Hz, ^14^CH), 7.669 (*s*, 1H, ^7^CH), 7.632–7.615 (*d*, 1H, *J* = 8.4 Hz, ^2^CH), 7.564–7.530 (*t*, 1H, *J* = 8.4 Hz, ^3^CH), 7.526–7.510 (*d*, 1H, *J* = 8.4 Hz, ^5^CH), 7.507–7.474 (*t*, 1H, *J* = 8.4 Hz, ^4^CH), 7.407–7.355 (*m*, 2H, ^15^CH, ^16^CH), 5.595 (*s*, 2H, ^10^CH_2_). ^13^C NMR (125 MHz, CDCl_3_) in ppm: 183.38 (C9), 164.79 (C11), 155.69 (C1), 150.41 (C8), 134.23 (C12), 133.09 (C15), 132.03 (C16), 131.20 (C3), 129.01 (C6), 128.81 (C14), 126.71 (C5), 126.70 (C4), 124.25 (C13), 123.55 (C17), 113.57 (C7), 112.51 (C2), 66.43 (C10). FT–IR (ATR (solid) cm^−1^): 3074 (Ar C—H, ν), 2949 (C—H, ν), 1736, 1686 (C=O, ν), 1612 (C=C, ν), 1554, 1472 (Ar C=C, ν), 1255, 1115 (C—O, ν), 1066 (C—Cl, ν).

**2-(Benzo­furan-2-yl)-2-oxoethyl 2-meth­oxy­benzoate (II):**

Solvent used to grow crystals: acetone; yield: 83%; m.p. 378–380 K. ^1^H NMR (500 MHz, CDCl_3_) in ppm: δ 8.047–8.032 (*d*, 1H, *J* = 8.0 Hz, ^17^CH), 7.768–7.752 (*d*, 1H, *J* = 8.0 Hz, ^14^CH), 7.681 (*s*, 1H, ^7^CH), 7.636–7.619 (*d*, 1H, *J* = 8.5 Hz, ^2^CH), 7.569–7.523 (*m*, 2H, ^5^CH, ^16^CH), 7.381–7.349 (t, 1H, *J* = 8.0 Hz, ^15^CH), 7.071–7.033 (*m*, 2H,3CH, ^4^CH), 5.535 (*s*, 2H, ^10^CH_2_), 3.955 (*s*, 3H, ^18^CH_3_). ^13^C NMR (125 MHz, CDCl_3_) in ppm: 183.98 (C9), 165.18 (C11), 159.68 (C13), 155.66 (C1), 150.55 (C8), 134.22 (C15), 132.33 (C17), 128.68 (C3), 126.77 (C6), 124.15 (C5), 123.48 (C4), 120.26 (C16), 118.80 (C12), 113.57 (C7), 112.53 (C2), 112.10 (C14), 66.09 (C10), 56.05 (C18). FT–IR (ATR (solid) cm^−1^): 3081 (Ar C—H, ν), 2921 (C—H, ν), 1762, 1686 (C=O, ν), 1601 (C=C, ν), 1554, 1465 (Ar C=C, ν), 1255, 1101 (C—O, ν).

## Refinement   

Crystal data, data collection and structure refinement details are tabulated in Table 3 ▸. All C-bound H atoms were positioned geometrically (C—H = 0.93–0.97 Å). Refinement was done using a riding model with *U*_iso_(H) = 1.5*U*_eq_(C-meth­yl) and 1.2*U*_eq_(C) for other H atoms.

## Supplementary Material

Crystal structure: contains datablock(s) global, I, II. DOI: 10.1107/S2056989017010556/qm2117sup1.cif

Structure factors: contains datablock(s) I. DOI: 10.1107/S2056989017010556/qm2117Isup2.hkl

Structure factors: contains datablock(s) II. DOI: 10.1107/S2056989017010556/qm2117IIsup3.hkl

CCDC references: 1449585, 1037757

Additional supporting information:  crystallographic information; 3D view; checkCIF report

## Figures and Tables

**Table 1:** Hydrogen-bond geometry (Å, °) for (I)

*D*—H⋯*A*	*D*—H	H⋯*A*	*D*⋯*A*	*D*—H⋯*A*
C10—H10*B*⋯O4^i^	0.97	2.53	3.495 (2)	176

Symmetry code: (i) 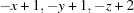.

**Table 2:** Hydrogen-bond geometry (Å, °) for (II) *Cg*3 is the centroid of the C12–C17 ring.

*D*—H⋯*A*	*D*—H	H⋯*A*	*D*⋯*A*	*D*—H⋯*A*
C2—H2*A*⋯O3^i^	0.95	2.45	3.2677 (15)	145
C7—H7*A*⋯O4^ii^	0.95	2.31	3.2352 (16)	163
C10—H10*A*⋯O3^iii^	0.99	2.55	3.3746 (14)	141
C10—H10*B*⋯O4^ii^	0.99	2.44	3.2028 (17)	134
C17—H17*A*⋯O1^iii^	0.95	2.55	3.2730 (15)	134
C18—H18*C*⋯*Cg*3^iv^	0.98	2.81	3.6181 (16)	141

Symmetry codes: (i) 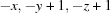; (ii) 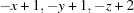; (iii) 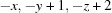; (iv) 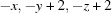.

**Table 3:** Experimental details

	(I)	(II)
Crystal data		
Chemical formula	C_17_H_11_ClO_4_	C_18_H_14_O_5_
*M*_r_	314.71	310.29
Crystal system, space group	Triclinic, *P* 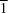	Triclinic, *P* 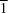
Temperature (K)	294	100
*a*, *b*, *c* (Å)	5.5333 (8), 11.3212 (17), 11.5186 (18)	7.4094 (3), 9.7566 (4), 10.5832 (5)
α, β, γ (°)	92.283 (3), 91.536 (3), 99.638 (3)	83.430 (1), 71.808 (1), 87.265 (1)
*V* (Å^3^)	710.41 (19)	721.99 (5)
*Z*	2	2
Radiation type	Mo *K*α	Mo *K*α
μ (mm^−1^)	0.28	0.11
Crystal size (mm)	0.54 × 0.25 × 0.21	0.51 × 0.35 × 0.11

Data collection		
Diffractometer	Bruker APEXII DUO CCD area-detector	Bruker APEXII DUO CCD area-detector
Absorption correction	Multi-scan (*SADABS*; Bruker, 2009 ▸)	Multi-scan (*SADABS*; Krause *et al.*, 2015 ▸)
*T*_min_, *T*_max_	0.799, 0.944	0.766, 0.950
No. of measured, independent and observed [*I* > 2σ(*I*)] reflections	12081, 3798, 2860	27838, 4286, 3615
*R*_int_	0.026	0.046
(sin θ/λ)_max_ (Å^−1^)	0.688	0.708

Refinement		
*R*[*F* ^2^ > 2σ(*F* ^2^)], *wR*(*F* ^2^), *S*	0.045, 0.145, 1.03	0.045, 0.124, 1.04
No. of reflections	3798	4286
No. of parameters	199	209
H-atom treatment	H-atom parameters constrained	H-atom parameters constrained
Δρ_max_, Δρ_min_ (e Å^−3^)	0.31, −0.27	0.52, −0.33

Computer programs: *APEX2* and *SAINT* (Bruker, 2009 ▸), *XS* (Sheldrick, 2013 ▸), *SHELXL2013* (Sheldrick, 2015 ▸), *Mercury* (Macrae *et al.*, 2006 ▸) and *PLATON* (Spek, 2009 ▸).

## supplementary crystallographic information

### 2-(Benzofuran-2-yl)-2-oxoethyl 2-chlorobenzoate (I) Crystal data

**Table:**

C_17_H_11_ClO_4_	*Z* = 2
*M**_r_* = 314.71	*F*(000) = 324
Triclinic, *P*1	*D*_x_ = 1.471 Mg m^−^^3^
*a* = 5.5333 (8) Å	Mo *K*α radiation, λ = 0.71073 Å
*b* = 11.3212 (17) Å	Cell parameters from 4675 reflections
*c* = 11.5186 (18) Å	θ = 2.5–29.0°
α = 92.283 (3)°	µ = 0.28 mm^−^^1^
β = 91.536 (3)°	*T* = 294 K
γ = 99.638 (3)°	Block, yellow
*V* = 710.41 (19) Å^3^	0.54 × 0.25 × 0.21 mm

### 2-(Benzofuran-2-yl)-2-oxoethyl 2-chlorobenzoate (I) Data collection

**Table:**

Bruker APEXII DUO CCD area-detector diffractometer	3798 independent reflections
Radiation source: fine-focus sealed tube	2860 reflections with *I* > 2σ(*I*)
Graphite monochromator	*R*_int_ = 0.026
φ and ω scans	θ_max_ = 29.3°, θ_min_ = 1.8°
Absorption correction: multi-scan (SADABS; Bruker, 2009)	*h* = −7→7
*T*_min_ = 0.799, *T*_max_ = 0.944	*k* = −15→15
12081 measured reflections	*l* = −15→15

### 2-(Benzofuran-2-yl)-2-oxoethyl 2-chlorobenzoate (I) Refinement

**Table:**

Refinement on *F*^2^	0 restraints
Least-squares matrix: full	Hydrogen site location: inferred from neighbouring sites
*R*[*F*^2^ > 2σ(*F*^2^)] = 0.045	H-atom parameters constrained
*wR*(*F*^2^) = 0.145	*w* = 1/[σ^2^(*F*_o_^2^) + (0.0759*P*)^2^ + 0.1547*P*] where *P* = (*F*_o_^2^ + 2*F*_c_^2^)/3
*S* = 1.03	(Δ/σ)_max_ = 0.001
3798 reflections	Δρ_max_ = 0.31 e Å^−^^3^
199 parameters	Δρ_min_ = −0.27 e Å^−^^3^

### 2-(Benzofuran-2-yl)-2-oxoethyl 2-chlorobenzoate (I) Special details

**Table:**

Geometry. All esds (except the esd in the dihedral angle between two l.s. planes) are estimated using the full covariance matrix. The cell esds are taken into account individually in the estimation of esds in distances, angles and torsion angles; correlations between esds in cell parameters are only used when they are defined by crystal symmetry. An approximate (isotropic) treatment of cell esds is used for estimating esds involving l.s. planes.

### 2-(Benzofuran-2-yl)-2-oxoethyl 2-chlorobenzoate (I) Fractional atomic coordinates and isotropic or equivalent isotropic displacement parameters (Å^2^)

**Table:**

	*x*	*y*	*z*	*U*_iso_*/*U*_eq_	
Cl1	1.19628 (9)	0.69545 (5)	1.14636 (5)	0.06498 (18)	
O1	0.7395 (2)	0.47462 (10)	0.60559 (10)	0.0487 (3)	
O2	0.5749 (2)	0.74445 (10)	0.91565 (10)	0.0472 (3)	
O3	0.8481 (3)	0.67643 (12)	0.74537 (13)	0.0669 (4)	
O4	0.7470 (3)	0.61109 (11)	1.01203 (13)	0.0651 (4)	
C1	0.6380 (3)	0.36403 (15)	0.55782 (13)	0.0451 (4)	
C2	0.7437 (4)	0.30006 (18)	0.47427 (16)	0.0595 (5)	
H2A	0.8925	0.3303	0.4421	0.071*	
C3	0.6141 (5)	0.18868 (19)	0.44183 (18)	0.0683 (6)	
H3A	0.6774	0.1419	0.3859	0.082*	
C4	0.3920 (5)	0.14375 (18)	0.48973 (18)	0.0653 (5)	
H4A	0.3119	0.0673	0.4662	0.078*	
C5	0.2876 (4)	0.20964 (17)	0.57136 (17)	0.0579 (4)	
H5A	0.1373	0.1796	0.6023	0.069*	
C6	0.4152 (3)	0.32335 (14)	0.60635 (13)	0.0443 (4)	
C7	0.3768 (3)	0.41575 (15)	0.68858 (14)	0.0450 (4)	
H7A	0.2418	0.4159	0.7349	0.054*	
C8	0.5753 (3)	0.50274 (14)	0.68559 (13)	0.0429 (3)	
C9	0.6496 (3)	0.61413 (14)	0.75465 (14)	0.0459 (4)	
C10	0.4630 (3)	0.64691 (15)	0.83864 (15)	0.0489 (4)	
H10A	0.3262	0.6698	0.7957	0.059*	
H10B	0.4008	0.5781	0.8832	0.059*	
C11	0.7212 (3)	0.71241 (14)	1.00006 (14)	0.0438 (3)	
C12	0.8368 (3)	0.81814 (14)	1.07463 (13)	0.0419 (3)	
C13	1.0492 (3)	0.81829 (16)	1.14281 (14)	0.0465 (4)	
C14	1.1527 (4)	0.9200 (2)	1.20965 (16)	0.0601 (5)	
H14A	1.2950	0.9196	1.2544	0.072*	
C15	1.0459 (4)	1.02112 (19)	1.20989 (17)	0.0638 (5)	
H15A	1.1165	1.0892	1.2543	0.077*	
C16	0.8358 (4)	1.02186 (17)	1.14495 (17)	0.0619 (5)	
H16A	0.7621	1.0899	1.1461	0.074*	
C17	0.7331 (4)	0.92144 (15)	1.07765 (16)	0.0515 (4)	
H17A	0.5910	0.9232	1.0333	0.062*	

### 2-(Benzofuran-2-yl)-2-oxoethyl 2-chlorobenzoate (I) Atomic displacement parameters (Å^2^)

**Table:**

	*U*^11^	*U*^22^	*U*^33^	*U*^12^	*U*^13^	*U*^23^
Cl1	0.0480 (3)	0.0749 (3)	0.0767 (3)	0.0207 (2)	−0.0007 (2)	0.0182 (2)
O1	0.0479 (6)	0.0484 (6)	0.0486 (6)	0.0049 (5)	0.0077 (5)	−0.0005 (5)
O2	0.0540 (7)	0.0423 (6)	0.0454 (6)	0.0109 (5)	−0.0056 (5)	−0.0029 (4)
O3	0.0618 (8)	0.0570 (8)	0.0754 (9)	−0.0079 (7)	0.0171 (7)	−0.0119 (6)
O4	0.0736 (9)	0.0398 (6)	0.0813 (9)	0.0112 (6)	−0.0169 (7)	0.0035 (6)
C1	0.0504 (9)	0.0461 (8)	0.0401 (7)	0.0120 (7)	−0.0007 (6)	0.0030 (6)
C2	0.0661 (12)	0.0651 (12)	0.0506 (9)	0.0201 (10)	0.0100 (8)	−0.0007 (8)
C3	0.0932 (16)	0.0647 (12)	0.0519 (10)	0.0311 (12)	0.0006 (10)	−0.0109 (9)
C4	0.0868 (15)	0.0502 (10)	0.0568 (10)	0.0101 (10)	−0.0124 (10)	−0.0096 (8)
C5	0.0612 (11)	0.0530 (10)	0.0554 (10)	0.0001 (8)	−0.0054 (8)	−0.0018 (8)
C6	0.0471 (9)	0.0457 (8)	0.0402 (7)	0.0090 (7)	−0.0036 (6)	0.0019 (6)
C7	0.0427 (8)	0.0485 (9)	0.0435 (8)	0.0071 (7)	0.0015 (6)	0.0000 (6)
C8	0.0449 (8)	0.0439 (8)	0.0405 (7)	0.0096 (6)	0.0019 (6)	0.0022 (6)
C9	0.0478 (9)	0.0426 (8)	0.0466 (8)	0.0053 (7)	0.0010 (7)	0.0020 (6)
C10	0.0497 (9)	0.0476 (9)	0.0480 (8)	0.0072 (7)	−0.0030 (7)	−0.0073 (7)
C11	0.0437 (8)	0.0413 (8)	0.0473 (8)	0.0093 (6)	0.0020 (6)	0.0037 (6)
C12	0.0425 (8)	0.0422 (8)	0.0407 (7)	0.0060 (6)	0.0035 (6)	0.0039 (6)
C13	0.0404 (8)	0.0553 (9)	0.0438 (8)	0.0056 (7)	0.0057 (6)	0.0091 (7)
C14	0.0514 (10)	0.0745 (13)	0.0478 (9)	−0.0070 (9)	−0.0029 (8)	0.0010 (8)
C15	0.0749 (13)	0.0563 (11)	0.0528 (10)	−0.0084 (10)	0.0052 (9)	−0.0070 (8)
C16	0.0786 (14)	0.0459 (10)	0.0604 (11)	0.0097 (9)	0.0077 (10)	−0.0062 (8)
C17	0.0557 (10)	0.0446 (9)	0.0548 (9)	0.0116 (7)	−0.0016 (8)	−0.0009 (7)

### 2-(Benzofuran-2-yl)-2-oxoethyl 2-chlorobenzoate (I) Geometric parameters (Å, º)

**Table:**

Cl1—C13	1.7261 (18)	C7—C8	1.349 (2)
O1—C1	1.371 (2)	C7—H7A	0.9300
O1—C8	1.3764 (19)	C8—C9	1.456 (2)
O2—C11	1.3480 (19)	C9—C10	1.514 (2)
O2—C10	1.4328 (19)	C10—H10A	0.9700
O3—C9	1.212 (2)	C10—H10B	0.9700
O4—C11	1.1922 (19)	C11—C12	1.487 (2)
C1—C2	1.382 (2)	C12—C17	1.386 (2)
C1—C6	1.382 (2)	C12—C13	1.396 (2)
C2—C3	1.374 (3)	C13—C14	1.390 (3)
C2—H2A	0.9300	C14—C15	1.373 (3)
C3—C4	1.386 (4)	C14—H14A	0.9300
C3—H3A	0.9300	C15—C16	1.367 (3)
C4—C5	1.375 (3)	C15—H15A	0.9300
C4—H4A	0.9300	C16—C17	1.381 (3)
C5—C6	1.399 (2)	C16—H16A	0.9300
C5—H5A	0.9300	C17—H17A	0.9300
C6—C7	1.429 (2)		
			
C1—O1—C8	105.45 (12)	C8—C9—C10	115.69 (15)
C11—O2—C10	114.00 (12)	O2—C10—C9	109.79 (14)
O1—C1—C2	125.23 (17)	O2—C10—H10A	109.7
O1—C1—C6	110.63 (14)	C9—C10—H10A	109.7
C2—C1—C6	124.13 (17)	O2—C10—H10B	109.7
C3—C2—C1	115.5 (2)	C9—C10—H10B	109.7
C3—C2—H2A	122.2	H10A—C10—H10B	108.2
C1—C2—H2A	122.2	O4—C11—O2	122.75 (16)
C2—C3—C4	122.16 (19)	O4—C11—C12	126.00 (16)
C2—C3—H3A	118.9	O2—C11—C12	111.24 (13)
C4—C3—H3A	118.9	C17—C12—C13	117.76 (16)
C5—C4—C3	121.47 (19)	C17—C12—C11	119.77 (15)
C5—C4—H4A	119.3	C13—C12—C11	122.48 (14)
C3—C4—H4A	119.3	C14—C13—C12	120.36 (17)
C4—C5—C6	117.8 (2)	C14—C13—Cl1	117.41 (14)
C4—C5—H5A	121.1	C12—C13—Cl1	122.23 (14)
C6—C5—H5A	121.1	C15—C14—C13	120.29 (18)
C1—C6—C5	118.95 (16)	C15—C14—H14A	119.9
C1—C6—C7	105.80 (15)	C13—C14—H14A	119.9
C5—C6—C7	135.22 (17)	C16—C15—C14	120.08 (18)
C8—C7—C6	106.35 (15)	C16—C15—H15A	120.0
C8—C7—H7A	126.8	C14—C15—H15A	120.0
C6—C7—H7A	126.8	C15—C16—C17	119.91 (19)
C7—C8—O1	111.77 (14)	C15—C16—H16A	120.0
C7—C8—C9	131.98 (15)	C17—C16—H16A	120.0
O1—C8—C9	116.14 (14)	C16—C17—C12	121.59 (18)
O3—C9—C8	122.07 (16)	C16—C17—H17A	119.2
O3—C9—C10	122.24 (15)	C12—C17—H17A	119.2
			
C8—O1—C1—C2	−178.83 (16)	O1—C8—C9—C10	177.42 (13)
C8—O1—C1—C6	0.04 (16)	C11—O2—C10—C9	−76.19 (17)
O1—C1—C2—C3	177.55 (16)	O3—C9—C10—O2	−10.9 (2)
C6—C1—C2—C3	−1.2 (3)	C8—C9—C10—O2	169.07 (13)
C1—C2—C3—C4	0.1 (3)	C10—O2—C11—O4	−2.9 (2)
C2—C3—C4—C5	1.1 (3)	C10—O2—C11—C12	178.34 (13)
C3—C4—C5—C6	−1.1 (3)	O4—C11—C12—C17	−158.19 (18)
O1—C1—C6—C5	−177.75 (14)	O2—C11—C12—C17	20.6 (2)
C2—C1—C6—C5	1.1 (2)	O4—C11—C12—C13	22.0 (3)
O1—C1—C6—C7	0.55 (17)	O2—C11—C12—C13	−159.28 (14)
C2—C1—C6—C7	179.44 (16)	C17—C12—C13—C14	−0.9 (2)
C4—C5—C6—C1	0.0 (2)	C11—C12—C13—C14	178.94 (15)
C4—C5—C6—C7	−177.63 (18)	C17—C12—C13—Cl1	−179.83 (13)
C1—C6—C7—C8	−0.93 (17)	C11—C12—C13—Cl1	0.0 (2)
C5—C6—C7—C8	176.95 (18)	C12—C13—C14—C15	0.5 (3)
C6—C7—C8—O1	1.01 (17)	Cl1—C13—C14—C15	179.46 (15)
C6—C7—C8—C9	−175.02 (16)	C13—C14—C15—C16	0.5 (3)
C1—O1—C8—C7	−0.67 (17)	C14—C15—C16—C17	−1.0 (3)
C1—O1—C8—C9	176.04 (13)	C15—C16—C17—C12	0.6 (3)
C7—C8—C9—O3	173.32 (18)	C13—C12—C17—C16	0.4 (3)
O1—C8—C9—O3	−2.6 (2)	C11—C12—C17—C16	−179.46 (17)
C7—C8—C9—C10	−6.7 (3)		

### 2-(Benzofuran-2-yl)-2-oxoethyl 2-chlorobenzoate (I) Hydrogen-bond geometry (Å, º)

**Table:**

*D*—H···*A*	*D*—H	H···*A*	*D*···*A*	*D*—H···*A*
C10—H10*B*···O4^i^	0.97	2.53	3.495 (2)	176
C17—H17*A*···O2	0.93	2.38	2.700 (2)	100

Symmetry code: (i) −*x*+1, −*y*+1, −*z*+2.

### 2-(Benzofuran-2-yl)-2-oxoethyl 2-methoxybenzoate (II) Crystal data

**Table:**

C_18_H_14_O_5_	*Z* = 2
*M**_r_* = 310.29	*F*(000) = 324
Triclinic, *P*1	*D*_x_ = 1.427 Mg m^−^^3^
*a* = 7.4094 (3) Å	Mo *K*α radiation, λ = 0.71073 Å
*b* = 9.7566 (4) Å	Cell parameters from 8829 reflections
*c* = 10.5832 (5) Å	θ = 2.8–30.2°
α = 83.430 (1)°	µ = 0.11 mm^−^^1^
β = 71.808 (1)°	*T* = 100 K
γ = 87.265 (1)°	Block, colourless
*V* = 721.99 (5) Å^3^	0.51 × 0.35 × 0.11 mm

### 2-(Benzofuran-2-yl)-2-oxoethyl 2-methoxybenzoate (II) Data collection

**Table:**

Bruker APEXII DUO CCD area-detector diffractometer	4286 independent reflections
Radiation source: fine-focus sealed tube	3615 reflections with *I* > 2σ(*I*)
Graphite monochromator	*R*_int_ = 0.046
φ and ω scans	θ_max_ = 30.2°, θ_min_ = 2.0°
Absorption correction: multi-scan (SADABS; Krause *et al.*, 2015)	*h* = −10→10
*T*_min_ = 0.766, *T*_max_ = 0.950	*k* = −13→13
27838 measured reflections	*l* = −14→14

### 2-(Benzofuran-2-yl)-2-oxoethyl 2-methoxybenzoate (II) Refinement

**Table:**

Refinement on *F*^2^	0 restraints
Least-squares matrix: full	Hydrogen site location: inferred from neighbouring sites
*R*[*F*^2^ > 2σ(*F*^2^)] = 0.045	H-atom parameters constrained
*wR*(*F*^2^) = 0.124	*w* = 1/[σ^2^(*F*_o_^2^) + (0.064*P*)^2^ + 0.3014*P*] where *P* = (*F*_o_^2^ + 2*F*_c_^2^)/3
*S* = 1.04	(Δ/σ)_max_ = 0.001
4286 reflections	Δρ_max_ = 0.52 e Å^−^^3^
209 parameters	Δρ_min_ = −0.33 e Å^−^^3^

### 2-(Benzofuran-2-yl)-2-oxoethyl 2-methoxybenzoate (II) Special details

**Table:**

Geometry. All esds (except the esd in the dihedral angle between two l.s. planes) are estimated using the full covariance matrix. The cell esds are taken into account individually in the estimation of esds in distances, angles and torsion angles; correlations between esds in cell parameters are only used when they are defined by crystal symmetry. An approximate (isotropic) treatment of cell esds is used for estimating esds involving l.s. planes.

### 2-(Benzofuran-2-yl)-2-oxoethyl 2-methoxybenzoate (II) Fractional atomic coordinates and isotropic or equivalent isotropic displacement parameters (Å^2^)

**Table:**

	*x*	*y*	*z*	*U*_iso_*/*U*_eq_	
O1	0.17733 (11)	0.47109 (8)	0.59014 (8)	0.01564 (17)	
O2	0.17204 (12)	0.66894 (8)	0.98339 (8)	0.01707 (18)	
O3	0.01605 (12)	0.62351 (9)	0.79953 (8)	0.01888 (18)	
O4	0.36010 (15)	0.60784 (11)	1.11020 (10)	0.0314 (2)	
O5	0.30167 (13)	0.95091 (9)	0.94102 (8)	0.01990 (19)	
C1	0.28755 (15)	0.38063 (11)	0.50676 (11)	0.0145 (2)	
C2	0.26141 (17)	0.34956 (13)	0.38921 (11)	0.0190 (2)	
H2A	0.1620	0.3901	0.3580	0.023*	
C3	0.38996 (18)	0.25537 (13)	0.32014 (12)	0.0215 (2)	
H3A	0.3782	0.2302	0.2390	0.026*	
C4	0.53699 (18)	0.19608 (13)	0.36640 (12)	0.0224 (2)	
H4A	0.6229	0.1326	0.3157	0.027*	
C5	0.55911 (17)	0.22858 (13)	0.48483 (12)	0.0198 (2)	
H5A	0.6583	0.1878	0.5162	0.024*	
C6	0.43110 (15)	0.32313 (11)	0.55666 (11)	0.0146 (2)	
C7	0.40703 (16)	0.38347 (11)	0.67825 (11)	0.0149 (2)	
H7A	0.4830	0.3667	0.7362	0.018*	
C8	0.25361 (15)	0.46946 (11)	0.69393 (10)	0.0142 (2)	
C9	0.15814 (15)	0.55579 (11)	0.79978 (11)	0.0142 (2)	
C10	0.24468 (16)	0.55128 (11)	0.91227 (11)	0.0151 (2)	
H10A	0.2093	0.4650	0.9728	0.018*	
H10B	0.3849	0.5548	0.8758	0.018*	
C11	0.24999 (15)	0.68820 (12)	1.07844 (11)	0.0153 (2)	
C12	0.17567 (15)	0.81458 (11)	1.14463 (11)	0.0142 (2)	
C13	0.19740 (15)	0.94584 (12)	1.07249 (11)	0.0153 (2)	
C14	0.11950 (16)	1.06035 (12)	1.13868 (12)	0.0180 (2)	
H14A	0.1303	1.1496	1.0906	0.022*	
C15	0.02584 (17)	1.04355 (13)	1.27532 (12)	0.0206 (2)	
H15A	−0.0280	1.1220	1.3198	0.025*	
C16	0.00940 (18)	0.91521 (13)	1.34781 (12)	0.0212 (2)	
H16A	−0.0537	0.9054	1.4414	0.025*	
C17	0.08654 (17)	0.80078 (12)	1.28184 (11)	0.0180 (2)	
H17A	0.0782	0.7123	1.3310	0.022*	
C18	0.33754 (19)	1.08448 (13)	0.86819 (12)	0.0226 (2)	
H18A	0.4241	1.0758	0.7780	0.034*	
H18B	0.3955	1.1423	0.9145	0.034*	
H18C	0.2176	1.1270	0.8621	0.034*	

### 2-(Benzofuran-2-yl)-2-oxoethyl 2-methoxybenzoate (II) Atomic displacement parameters (Å^2^)

**Table:**

	*U*^11^	*U*^22^	*U*^33^	*U*^12^	*U*^13^	*U*^23^
O1	0.0172 (4)	0.0179 (4)	0.0137 (4)	0.0017 (3)	−0.0075 (3)	−0.0026 (3)
O2	0.0213 (4)	0.0164 (4)	0.0176 (4)	0.0062 (3)	−0.0112 (3)	−0.0062 (3)
O3	0.0184 (4)	0.0207 (4)	0.0198 (4)	0.0047 (3)	−0.0093 (3)	−0.0037 (3)
O4	0.0403 (6)	0.0307 (5)	0.0362 (5)	0.0198 (4)	−0.0288 (5)	−0.0166 (4)
O5	0.0265 (4)	0.0163 (4)	0.0143 (4)	−0.0002 (3)	−0.0034 (3)	0.0007 (3)
C1	0.0155 (5)	0.0152 (5)	0.0129 (5)	−0.0015 (4)	−0.0046 (4)	−0.0013 (4)
C2	0.0209 (5)	0.0230 (6)	0.0147 (5)	−0.0040 (4)	−0.0075 (4)	−0.0013 (4)
C3	0.0237 (6)	0.0262 (6)	0.0153 (5)	−0.0050 (5)	−0.0053 (4)	−0.0056 (4)
C4	0.0234 (6)	0.0234 (6)	0.0201 (6)	0.0007 (4)	−0.0041 (4)	−0.0086 (4)
C5	0.0197 (5)	0.0202 (5)	0.0201 (5)	0.0025 (4)	−0.0065 (4)	−0.0046 (4)
C6	0.0167 (5)	0.0141 (5)	0.0129 (5)	−0.0006 (4)	−0.0048 (4)	−0.0007 (4)
C7	0.0180 (5)	0.0147 (5)	0.0128 (5)	0.0001 (4)	−0.0063 (4)	−0.0004 (4)
C8	0.0165 (5)	0.0146 (5)	0.0125 (5)	−0.0009 (4)	−0.0062 (4)	−0.0005 (4)
C9	0.0161 (5)	0.0136 (5)	0.0133 (5)	−0.0012 (4)	−0.0053 (4)	−0.0003 (4)
C10	0.0184 (5)	0.0151 (5)	0.0132 (5)	0.0041 (4)	−0.0068 (4)	−0.0035 (4)
C11	0.0164 (5)	0.0163 (5)	0.0145 (5)	0.0012 (4)	−0.0070 (4)	−0.0017 (4)
C12	0.0155 (5)	0.0140 (5)	0.0144 (5)	0.0012 (4)	−0.0065 (4)	−0.0025 (4)
C13	0.0158 (5)	0.0166 (5)	0.0143 (5)	0.0006 (4)	−0.0061 (4)	−0.0014 (4)
C14	0.0198 (5)	0.0146 (5)	0.0206 (5)	0.0015 (4)	−0.0076 (4)	−0.0023 (4)
C15	0.0205 (5)	0.0193 (5)	0.0225 (6)	0.0013 (4)	−0.0059 (4)	−0.0072 (4)
C16	0.0231 (5)	0.0235 (6)	0.0153 (5)	−0.0011 (4)	−0.0023 (4)	−0.0050 (4)
C17	0.0207 (5)	0.0172 (5)	0.0160 (5)	−0.0018 (4)	−0.0056 (4)	−0.0008 (4)
C18	0.0278 (6)	0.0190 (5)	0.0197 (5)	−0.0041 (4)	−0.0072 (5)	0.0042 (4)

### 2-(Benzofuran-2-yl)-2-oxoethyl 2-methoxybenzoate (II) Geometric parameters (Å, º)

**Table:**

O1—C1	1.3726 (13)	C7—H7A	0.9500
O1—C8	1.3817 (13)	C8—C9	1.4591 (15)
O2—C11	1.3405 (13)	C9—C10	1.5141 (15)
O2—C10	1.4352 (13)	C10—H10A	0.9900
O3—C9	1.2165 (13)	C10—H10B	0.9900
O4—C11	1.2013 (14)	C11—C12	1.4854 (15)
O5—C13	1.3608 (13)	C12—C17	1.3885 (15)
O5—C18	1.4278 (14)	C12—C13	1.4029 (15)
C1—C2	1.3854 (15)	C13—C14	1.3918 (15)
C1—C6	1.3968 (15)	C14—C15	1.3891 (17)
C2—C3	1.3862 (17)	C14—H14A	0.9500
C2—H2A	0.9500	C15—C16	1.3811 (17)
C3—C4	1.4033 (18)	C15—H15A	0.9500
C3—H3A	0.9500	C16—C17	1.3888 (16)
C4—C5	1.3865 (17)	C16—H16A	0.9500
C4—H4A	0.9500	C17—H17A	0.9500
C5—C6	1.3983 (15)	C18—H18A	0.9800
C5—H5A	0.9500	C18—H18B	0.9800
C6—C7	1.4333 (15)	C18—H18C	0.9800
C7—C8	1.3589 (15)		
			
C1—O1—C8	105.39 (8)	C9—C10—H10A	110.3
C11—O2—C10	114.70 (8)	O2—C10—H10B	110.3
C13—O5—C18	116.85 (9)	C9—C10—H10B	110.3
O1—C1—C2	125.19 (10)	H10A—C10—H10B	108.5
O1—C1—C6	110.64 (9)	O4—C11—O2	123.18 (11)
C2—C1—C6	124.16 (11)	O4—C11—C12	124.35 (10)
C1—C2—C3	115.56 (11)	O2—C11—C12	112.37 (9)
C1—C2—H2A	122.2	C17—C12—C13	120.00 (10)
C3—C2—H2A	122.2	C17—C12—C11	118.36 (10)
C2—C3—C4	122.01 (11)	C13—C12—C11	121.63 (10)
C2—C3—H3A	119.0	O5—C13—C14	124.76 (10)
C4—C3—H3A	119.0	O5—C13—C12	115.95 (10)
C5—C4—C3	121.16 (11)	C14—C13—C12	119.25 (10)
C5—C4—H4A	119.4	C15—C14—C13	119.68 (11)
C3—C4—H4A	119.4	C15—C14—H14A	120.2
C4—C5—C6	118.01 (11)	C13—C14—H14A	120.2
C4—C5—H5A	121.0	C16—C15—C14	121.37 (11)
C6—C5—H5A	121.0	C16—C15—H15A	119.3
C1—C6—C5	119.09 (10)	C14—C15—H15A	119.3
C1—C6—C7	105.80 (9)	C15—C16—C17	119.02 (11)
C5—C6—C7	135.10 (11)	C15—C16—H16A	120.5
C8—C7—C6	106.09 (9)	C17—C16—H16A	120.5
C8—C7—H7A	127.0	C12—C17—C16	120.59 (11)
C6—C7—H7A	127.0	C12—C17—H17A	119.7
C7—C8—O1	112.07 (9)	C16—C17—H17A	119.7
C7—C8—C9	131.43 (10)	O5—C18—H18A	109.5
O1—C8—C9	116.50 (9)	O5—C18—H18B	109.5
O3—C9—C8	122.38 (10)	H18A—C18—H18B	109.5
O3—C9—C10	122.39 (10)	O5—C18—H18C	109.5
C8—C9—C10	115.20 (9)	H18A—C18—H18C	109.5
O2—C10—C9	107.13 (8)	H18B—C18—H18C	109.5
O2—C10—H10A	110.3		
			
C8—O1—C1—C2	179.76 (10)	C11—O2—C10—C9	−173.51 (9)
C8—O1—C1—C6	−0.15 (12)	O3—C9—C10—O2	−18.82 (14)
O1—C1—C2—C3	179.82 (10)	C8—C9—C10—O2	163.15 (9)
C6—C1—C2—C3	−0.28 (17)	C10—O2—C11—O4	−6.03 (17)
C1—C2—C3—C4	−0.22 (17)	C10—O2—C11—C12	177.51 (9)
C2—C3—C4—C5	0.57 (19)	O4—C11—C12—C17	−55.50 (17)
C3—C4—C5—C6	−0.42 (18)	O2—C11—C12—C17	120.91 (11)
O1—C1—C6—C5	−179.67 (10)	O4—C11—C12—C13	123.09 (14)
C2—C1—C6—C5	0.42 (17)	O2—C11—C12—C13	−60.51 (14)
O1—C1—C6—C7	−0.36 (12)	C18—O5—C13—C14	2.08 (16)
C2—C1—C6—C7	179.73 (10)	C18—O5—C13—C12	−175.45 (10)
C4—C5—C6—C1	−0.05 (17)	C17—C12—C13—O5	174.12 (10)
C4—C5—C6—C7	−179.11 (12)	C11—C12—C13—O5	−4.45 (15)
C1—C6—C7—C8	0.73 (12)	C17—C12—C13—C14	−3.55 (16)
C5—C6—C7—C8	179.88 (13)	C11—C12—C13—C14	177.88 (10)
C6—C7—C8—O1	−0.87 (12)	O5—C13—C14—C15	−175.85 (11)
C6—C7—C8—C9	178.16 (11)	C12—C13—C14—C15	1.60 (17)
C1—O1—C8—C7	0.65 (12)	C13—C14—C15—C16	0.55 (18)
C1—O1—C8—C9	−178.54 (9)	C14—C15—C16—C17	−0.74 (19)
C7—C8—C9—O3	−177.25 (11)	C13—C12—C17—C16	3.40 (17)
O1—C8—C9—O3	1.74 (16)	C11—C12—C17—C16	−177.99 (10)
C7—C8—C9—C10	0.77 (17)	C15—C16—C17—C12	−1.25 (18)
O1—C8—C9—C10	179.77 (9)		

### 2-(Benzofuran-2-yl)-2-oxoethyl 2-methoxybenzoate (II) Hydrogen-bond geometry (Å, º)

*Cg*3 is the centroid of the C12–C17 ring.

**Table:**

*D*—H···*A*	*D*—H	H···*A*	*D*···*A*	*D*—H···*A*
C2—H2*A*···O3^i^	0.95	2.45	3.2677 (15)	145
C7—H7*A*···O4^ii^	0.95	2.31	3.2352 (16)	163
C10—H10*A*···O3^iii^	0.99	2.55	3.3746 (14)	141
C10—H10*B*···O4^ii^	0.99	2.44	3.2028 (17)	134
C17—H17*A*···O1^iii^	0.95	2.55	3.2730 (15)	134
C18—H18*C*···*Cg*3^iv^	0.98	2.81	3.6181 (16)	141

Symmetry codes: (i) −*x*, −*y*+1, −*z*+1; (ii) −*x*+1, −*y*+1, −*z*+2; (iii) −*x*, −*y*+1, −*z*+2; (iv) −*x*, −*y*+2, −*z*+2.

### π–π interactions in compound (II).

**Table:**

Centroid 1	Centroid 2	Centroid-to-centroid distance (Å)	Symmetry code
*Cg*1	*Cg*1	3.4402 (7)	-x+1, -y+1, -z+1
*Cg*1	*Cg*2	3.6088 (7)	-x+1, -y+1, -z+1

*Cg*1 and *Cg*2 are the centroids of O1/C1/C6/C7/C8 and C1–C6 rings, respectively.
